# *ABCC2*-24C > T polymorphism is associated with the response to platinum/5-Fu-based neoadjuvant chemotherapy and better clinical outcomes in advanced gastric cancer patients

**DOI:** 10.18632/oncotarget.10961

**Published:** 2016-07-30

**Authors:** Ziyu Li, Xiaofang Xing, Fei Shan, Shuangxi Li, Zhongwu Li, Aitang Xiao, Zhaodong Xing, Kan Xue, Zhemin Li, Ying Hu, Yongning Jia, Rulin Miao, Lianhai Zhang, Zhaode Bu, Aiwen Wu, Jiafu Ji

**Affiliations:** ^1^ Department of Gastrointestinal Surgery, Key Laboratory of Carcinogenesis and Translational Research (Ministry of Education), Peking University Cancer Hospital and Institute, Beijing, China; ^2^ Department of Gastrointestinal Translational Research, Key Laboratory of Carcinogenesis and Translational Research (Ministry of Education), Peking University Cancer Hospital and Institute, Beijing, China; ^3^ Department of Pathology, Key Laboratory of Carcinogenesis and Translational Research (Ministry of Education), Peking University Cancer Hospital and Institute, Beijing, China; ^4^ Tissue Bank, Key Laboratory of Carcinogenesis and Translational Research (Ministry of Education), Peking University Cancer Hospital and Institute, Beijing, China

**Keywords:** ABCC2, rs717620, gastric cancer, neoadjuvant chemotherapy, overall survival

## Abstract

Several studies have evaluated the efficacy of neoadjuvant treatment using oxaliplatin and fluoropyrimidines in advanced gastric cancer (GC). However, preoperative biomarkers predictive of clinical outcome remain lacking. We examined polymorphisms in the *MTHFR, DPYD, UMPS, ABCB1, ABCC2, GSTP1, ERCC1,* and *XRCC1* genes to evaluate their usefulness as pharmacogenetic markers in a cohort of 103 GC patients treated with preoperative chemotherapy. DNA was extracted from peripheral blood cells, and the genotypes were analyzed using a SNaPShot^TM^ assay, polymerase chain reaction amplification, and sequencing. The *ABCC2*-24C > T (rs717620) genotype was associated with pathologic response to neoadjuvant chemotherapy. Patients with the TT and TC genotypes responded to neoadjuvant chemotherapy 3.80 times more often than those with the CC genotype (95% CI: 1.27–11.32). Patients with the CC genotype also had poorer outcomes than those with other genotypes. Thus, *ABCC2*-24C > T polymorphism may help to predict the response to preoperative chemotherapy in GC patients.

## INTRODUCTION

Gastric cancer (GC) is the third leading cause of cancer-related death in Eastern Asia and has the highest age-standardized incidence rate [1]. Fifty-eight percent of global GC deaths occur in China, Korea, and Japan. The most common treatments for GC are 5- fluorouracil (5-Fu) and platinum, though not all patients respond well to treatment. To determine which patients will benefit from this therapy, it is crucial to identify factors relevant to the 5-Fu/platinum response. Several commercial *in vitro* chemosensitivity tests are available [2]; however, suboptimal reproducibility, tumor cell heterogeneity, and poor correlation with clinical outcomes limit the efficacy of these assays.

Neoadjuvant chemotherapy (NAC) is also effective in down staging primary tumors, facilitating radical surgical resection, and treating systemic micrometastasis. Our previous studies demonstrated that treatment with FOLFOX NAC followed by radical surgery is safe and well-tolerated and improves survival in patients with resectable, locally advanced GC [3, 4]. Personalized medicine can help identify the most effective treatments and doses, while minimizing side effects and toxicity, for patients on an individual basis. However, additional indicators that can accurately predict prognoses and response to NAC drugs are needed to improve treatments.

In recent years, many studies have shown that single-nucleotide polymorphisms (SNPs) affect tumor occurrence and development and patient outcomes [5, 6]. For example, patients with fewer CYP2A6 rs3212986 variants responded better to cisplatin plus S-1 treatment and had longer overall survival [9]. Inter-individual variation in drug-metabolizing enzymes and the nucleotide excision repair (NER) system may affect anticancer drug efficacy by influencing DNA repair or related enzyme activities [7]. Additionally, Choi et al reported that genetic variations in genes that encode drug transporters influenced drug responses in patients treated with docetaxel [11]. Overexpression of MRP transporters and gankyrin contributes to arsenic trioxide resistance in liver and GC cells [13]. ATP binding cassette subfamily C member 2 (*ABCC2*), a member of the MRP transporter family that encodes multidrug resistance-associated protein 2, functions as an organic anion transporter [14]. The ABCC2-24C > T polymorphism increased responses to platinum-based chemotherapy in non-small cell lung cancer [15]. Many recent studies have also found that genes involved in DNA detoxification (e.g., glutathione S-transferases [*GSTs*] and excision repair cross-complementing 1 [*ERCC1*]) play a role in the effects of platinum, while methylene tetrahydrofolate reductase (*MTHFR*) and thymidylate synthase (*TS*) affect 5-Fu metabolism [8–10]. A meta-analysis revealed that polymorphisms in *ERCC1*, *GSTs*, *TS*, and *MTHFR* were closely associated with clinical outcomes in GC patients treated with platinum/5-FU-based chemotherapy [8]. X-ray repair cross-complementing protein 1 (*XRCC1*) polymorphisms were also associated with response to platinum-based NAC in cervical cancer patients [12].

In this study, we focused on a set of 23 SNPs, many of which are reportedly related to platinum/Fu-5-based chemotherapy resistance [7], from the Pharmacogenetics and Pharmacogenomics Knowledge Base (pharmGKB) database. We investigated associations between preoperative chemotherapy response and these SNP variants, which were located in the *MTHFR*, *DPYD* (dihydropyrimidine dehydrogenase), *UMPS* (uridine monophosphate synthetase), *ABCB1* (ATP binding cassette subfamily B member 1), *ABCC2*, *ERCC1*, *XRCC1*, and *GSTP1* (glutathione S-transferase pi 1) genes. Characterizing the value of these SNPs for predicting GC patient survival may help to improve personalized treatments.

## RESULTS

### Clinical findings

In total, 103 patients with stage II–III GC were recruited; 74 were males and 29 were females, reflecting the sex-stratified prevalence of GC in East Asia. Patient ages ranged from 26–75, with a mean of 58.3 (standard deviation [SD] ± 10.3) years. Every patient received 2–4 cycles of NAC prior to a D2 gastrectomy. Nineteen patients (18.5%) were treated with either FOLFOX6 or FOLFOX7 therapy, 46 patients (44.7%) with SOX therapy, 24 patients (23.2%) with XELOX therapy, 6 patients (5.8%) with an XP regimen, and 8 patients with radiation-based or paclitaxel and oxaliplatin preoperative therapy. Patient baseline clinical and tumor characteristics are listed in Table 1. Patients receiving NAC were classified based on tumor regression scores (TRG, according to the Becker's Criteria [16]). Eight patients had pathological complete responses (pCR) in the primary tumor, which is consistent with the postoperative pathological T0 (ypT0) stage. Of these 8 patients, 6 were classified as ypTNM pCR; the remaining 2 were not because they had positive lymph node involvement. Besides, all the patients were classified as responders (TGR score 1a or 1b) or non-responders (TGR score 2 or 3). No statistically significant differences in pCR rates were found among groups of patients receiving different NAC regimens.

**Table 1:** Clinical characteristics of the patients with gastric cancer

Variable	Patients (%)
**Gender**	
Male	74 (71.8)
Female	29 (28.2)
**Age (y)**	
Mean	58.3
Range	26–75
**Tumor location**	
EGJ	41 (39.8)
Body	27 (26.2)
Antrum	35 (34.0)
**Treatment**	
FOLFOX	19 (18.5)
SOX	46 (44.7)
XELOX	24 (23.3)
XP	6 (5.8)
Other	8 (7.7)
**Clinical tumor stage**	
II	33 (32.0)
III	70 (68.0)
**Tumor regression grade (TRG)**	
1a	8 (7.8)
1b	9 (8.7)
2	23 (22.3)
3	63 (61.2)

### Association between clinicopathological variables and OS

The 1-, 3-, and 5-year survival rates were 89.0%, 75.9%, and 64.7%, respectively. During the 5-year follow-up period, 33 of the 103 patients died (Supplementary Table S1). Clinicopathological characteristics, including pathological stage (pTNM), pathological T stage (pT), pathological N stage (pN), pathological M stage (pM), and vascular invasion, were associated with survival time (log-rank *p*-value < 0.05, Table 2). Furthermore, pathological stage was most strongly associated with OS after controlling for the experiment-wise type I error rate of 0.05 with the Bonferroni correction. The survival rate of TRG pCR (1a) patients was 100%, compared to 40.6% in patients with TRG scores of others (including 1b, 2 and 3). However, this difference did not reach statistical significance (*p* = 0.052). Sex, age, cancer location, surgery options, and differentiation were not significantly associated with survival time (*p* > 0.05, data not shown).

**Table 2:** Associations between the clinicopatholoigcal variables and overall survival

Factor	Patients (%)	Overall survival	*p*-value
**Clinical Stage**			0.732
II	33 (32.0)	0.666 ± 0.087	
III	70 (68.0)	0.590 ± 0.073	
**Pathological stage**			**0.000**
pCR	6 (5.8)	1.000 ± 0.000	
I	15 (14.5)	1.000 ± 0.000	
II	31 (30.1)	0.777 ± 0.081	
III	46 (44.7)	0.357 ± 0.103	
IV	5 (4.9)	0.000 ± 0.000	
**Pathological T stage**			**0.001**
0	8 (7.8)	1.000 ± 0.000	
1	4 (3.9)	1.000 ± 0.000	
2	15 (14.6)	0.929 ± 0.069	
3	33 (32.0)	0.509 ± 0.161	
4	43 (41.7)	0.418 ± 0.084	
**Pathological N stage**			**0.003**
0	40 (38.8)	0.830 ± 0.064	
1	27 (26.2)	0.662 ± 0.099	
2	12 (11.7)	0.417 ± 0.157	
3	24 (23.3)	0.000 ± 0.000	
**Pathological M stage**			**0.000**
0	5 (4.9)		
1	98 (95.1)		
**TRG**			0.052
pCR	8 (7.8)	1.000 ± 0.000	
others	95 (92.2)	0.406 ± 0.101	
**Vascular Invasion**			**0.003**
Negative	72 (69.9)	0.712 ± 0.058	
Positive	31 (30.1)	0.249 ± 0.183	
**Differentiation**			0.270
Well	2 (1.9)	0.500 ± 0.354	
Moderate	55 (53.4)	0.672 ± 0.076	
Poorly	46 (44.7)	0.560 ± 0.083	
**Rs717620**			**0.032**
CT + TT	39 (37.9)	0.758 ± 0.075	
CC	64 (62.1)	0.529 ± 0.077	

TRG: Tumor Regression Grade. pCR: pathological complete response.

### Quality control for the SNPs

Of the 23 SNPs examined, different variants were detected at 13 loci. The average genotyping success rate for these 13 loci was 99.8%: 1 data point was missing for the rs1045642 locus and 2 data points were missing for the rs3212986 locus. Furthermore, rs17376848 (*p* = 0.00139) and rs1695 (*p* = 0.000191) failed the HWE test after multi-test correction for an experiment-wise type I error rate of 0.05 (Table 3).

**Table 3:** Information for the single nucleotide polymorphism (SNPs) genotyped in this study

SNP^a^	CHR	Position	Alleles	Gene^b^	Amino Acid Translation	Genotype	Hardy-Weinberg Test^c^	Freq	Freq (CHB)^d^
rs1801131	1	11794419	C/A	*MTHFR*	Glu347Ala	2/17/84	0.271	0.102	0.202
rs1801133	1	11796321	C/T	*MTHFR*	Ala140Val	16/50/37	1.000	0.398	0.489
rs1801159	1	97515839	G/A	*DPYD*	Ile543Val	8/42/53	1.000	0.282	0.281
rs1801265	1	97883329	C/T	*DPYD*	Cys29Arg	3/15/85	0.064	0.102	0.060
rs1801160	1	97770920	A/G	*DPYD*	Val732Ile	0/2/101	1.000	0.010	0.015
rs17376848	1	97915624	G/A	*DPYD*	Phe632Phe	5/12/86	0.001*	0.107	0.143
rs2297595	1	98165091	G/A	*DPYD*	Met166Val	0/4/99	1.000	0.019	0.006
rs1801019	3	124456742	C/G	*UMPS*	Gly213Ala	4/28/71	0.503	0.175	0.136
rs1045642	7	87138645	T/C	*ABCB1*	Ile1145Ile	18/48/36	0.838	0.412	0.402
rs717620	10	101542578	T/C	*ABCC2*	5′UTR	3/36/64	0.556	0.204	0.214
rs3212986	11	45912736	T/G	*ERCC1*	Gln504Lys	12/48/41	0.830	0.356	0.329
rs1695	11	67352689	G/A	*GSTP1*	Ile105Val	1/57/45	0.000*	0.286	0.207
rs25487	19	44055726	A/G	*XRCC1*	Gln399Arg	9/33/61	0.183	0.248	0.253

a rs1801131 was excluded due to its susceptible allele frequency compared with the CHB reference; rs2297595 was a rare-variant locus; rs1737648 and rs1695 were excluded due to their departure from Hardy-Weinberg proportions.

b *MTHFR* (methylenetetrahydrofolate reductase); *MTHFR*, *DPYD* (dihydropyrimidine dehydrogenase), *UMPS* (uridine monophosphate synthetase), *ABCB1* (ATP binding cassette subfamily B member 1), *ABCC2*, *ERCC1*, *GSTP1* (glutathione S-transferase pi 1), and *XRCC1* (X-ray repair complementing defective repair in Chinese hamster cells 1).

c The Hardy-Weinberg test was conducted using Fisher's exact test. Loci that departed from the Hardy-Weinberg standard are marked by asterisks.

d Allele frequencies were extracted from HapMap/1 KG CHB samples.

The rs2297595 locus, with an allele frequency of 0.02, was a rare/low-frequency variant, consistent with its reported minor allele frequency of 0.006 in the Han Chinese in Beijing, China (CHB) cohort of the 1000 Genomes project (1KG). We also compared allele frequencies found in GC patients to those found in presumably cancer-free pseudo-controls from HapMap Chinese Han Samples (84 independent samples). The reference allele (C) for rs1801131 had a frequency of 0.102 in this study, compared to a frequency of 0.202 in the CHB cohort; a chi-square test comparing these frequencies yielded a *p*-value of 0.0078, indicating a potential quality issue in SNP calling. After stringent quality control, rs1801131, rs2297595, and rs1045642 were excluded from the analysis below (Table 3).

### Analysis of associations between rs717620, clinicopathological features, and survival

We further analyzed the association of each individual SNP with clinical features, particularly NAC efficacy and OS. TGR scores reflected the short-term efficacy of NAC, while OS reflected long-term efficacy. We also analyzed the association of each SNP with pathologic response and OS. The rs717620 locus was associated with TRG score. Under the dominance model, it had a *p*-value of 0.016 and an OR of 3.80 (95% CI: 1.27–11.32), indicating a protective role for the T allele (Table 4). No significant associations were observed between rs717620 and any of the following clinicopathological features: age, sex, clinical stage, pathological stage, and NAC regimen.

**Table 4:** Association between ABCC2-24C > T genotype and clinicopathologic variables

Variables	rs717620		OR^a^	95% CI	*P* value^b^
	TT/CT	CC			
**Age, years**					
< 60	16	31			
≥ 60	23	33	0.741	0.331–1.656	0.543
Sex					
Male	26	48			
Female	13	16	0.667	0.278–1.597	0.376
Clinical tumor stage					
II	14	19			
III	25	45	1.326	0.569–3.091	0.522
Pathological stage					
0	3	3			
1	6	9	1.500	0.223–10.077	
2	12	19	1.583	0.274–9.166	
3	15	31	2.067	0.372–11.483	
4	3	2	0.667	0.060–7.352	0.718
TGR					
Responders	11	6			
Nonresponders	28	58	3.798	1.274–11.320	0.016
Neoadjuvant chemotherapy regimen					
Folfox	9	10			
Sox	17	29	1.535	0.521–4.527	
Xelox	9	15	1.500	0.442–5.092	
XP	3	3	0.900	0.143–5.646	
other	1	7	6.300	0.644–61.631	0.517

OR, odds ratio; CI, confidence interval;

a Odds ratios comparing CC with TT/CT, including 95% confidence interval.

b Fisher's exact test.

**Figure 1:**
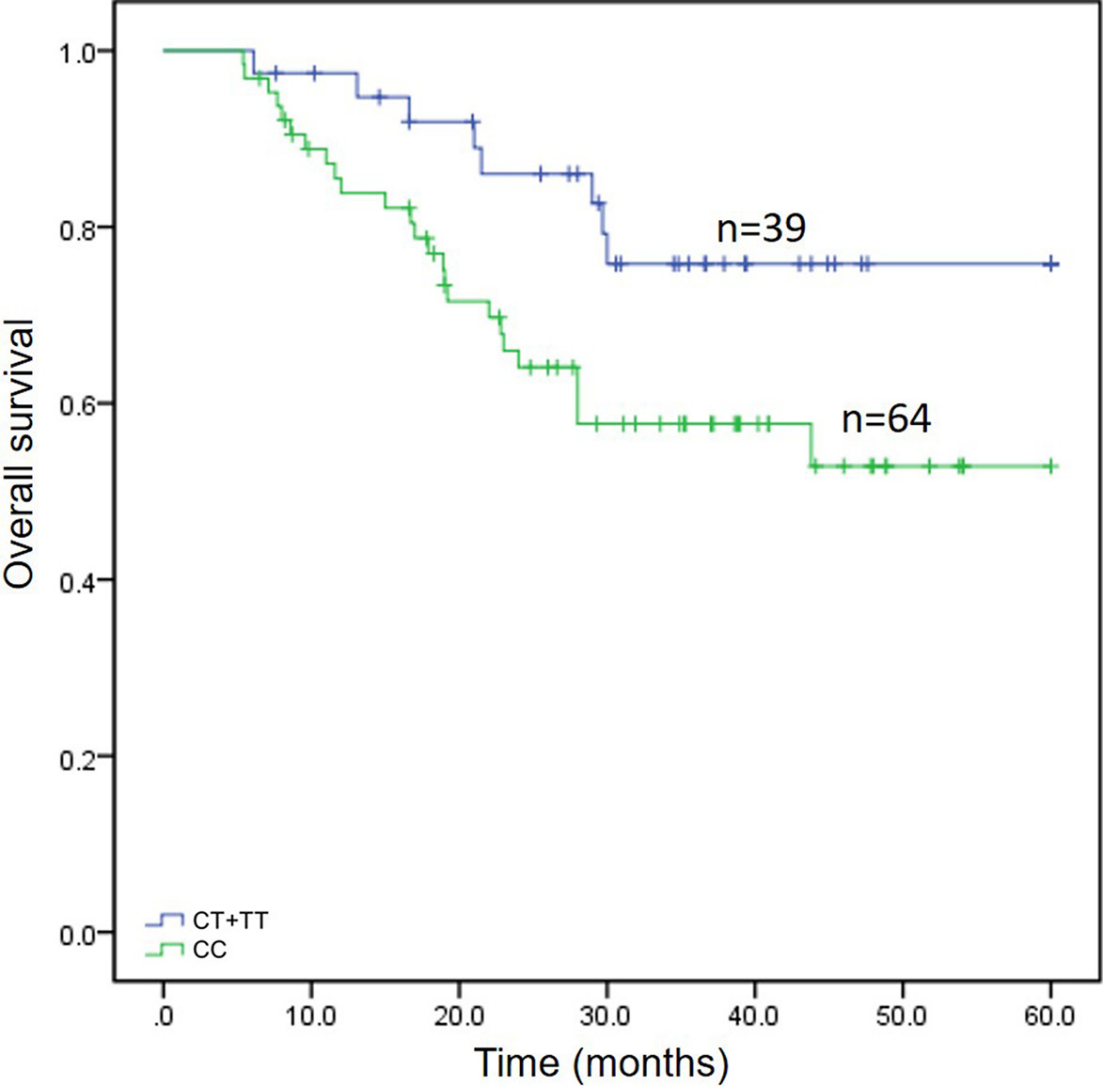
Survival analysis for 103 patients with gastric cancer depending on rs717620 status The horizontal axis indicates months of overall survival, and the vertical axis indicates survival of the 2 genotypic classes. Vertical hash marks indicate patient deaths.

In the survival analysis, rs717620 was associated with OS (Table 2). As shown in Figure 1, CC genotype patients comprised one group and TC or TT genotype patients a second group based on the rs717620 dominance model. The 39 TC/TT patients, 8 of whom died by the end of the study (OS, 0.758 ± 0.075), survived longer than the 64 CC patients, of whom 25 died (OS, 0.529 ± 0.077) (*p* = 0.032). TC/TT patients responded to NAC 3.798 times more often than CC patients and also had better prognoses. No other SNPs were significantly associated with NAC efficacy or OS (data not shown).

Pathological stage (pTNM), pathological T stage (pT), pathological N stage (pN), pathological M stage (pM), TRG, vascular invasion, and rs717620 were included in the multivariate Cox regression model. Pathological stage was the only independent prognostic factor (Table 5, HR: 6.316, 95% CI: 2.921–13.657, *p* = 0.000). Although TC or TT ABCC2 rs717620 genotype tended to have a protective role compared to the CC genotype (Table 5, HR: 0.454, 95% CI: 0.203–1.013), this effect did not reach statistical significance (*p* = 0.052).

**Table 5:** Multivariate analysis of survival in gastric cancer according to clinicopathologic factors and rs717620

Variables	HR	95% CI	*P* value
**pTNM**	6.316	2.291–13.657	0.000
**pN**	0.815	0.549–1.208	0.307
**rs717620**	0.454	0.203–1.013	0.054
**Vascular invastion**	0.823	0.363–1.864	0.640

## DISCUSSION

Despite its declining incidence in Western countries, gastric carcinoma remains the second most common cause of cancer-related mortality worldwide, largely due to its high incidence in East Asia [1]. Although some GC patients benefit from NAC, many do not respond or even progress during treatment. It is therefore important to identify biomarkers that predict individual resistance to NAC in the Chinese population. Recently, many inter-individual differences in drug response were found to be associated with alterations in genes that encode drug-metabolizing enzymes, transporters, or targets.

In this study, we investigated the association between clinicopathological features and OS in 103 Chinese GC patients recruited between 2008 and 2013 at Beijing Cancer Hospital. Survival analysis revealed that pathological stage, pathological T stage, pathological N stage, and vascular invasion were associated with OS. The eight patients with pCRs as defined by Becker's pathological criteria had the highest survival rate at 100%. Individuals who carry polymorphisms in certain genes may be resistant to chemoradiation therapies [7]. Therefore, we investigated the association of 13 SNPs in 8 genes with OS in the 103 GC patients. rs717620 polymorphisms were associated with TGR score and OS. The rs717620 *ABCC2*-24C > T genotype was associated with a pathologic response to NAC. Patients with TT or TC genotypes responded to NAC 3.798 times more often than those with the GG genotype. Furthermore, Patients with the CC genotype had poorer outcomes compared to the other genotypes. Genotype variations within the other SNPs examined were not associated with differences in survival or pathological response.

The *ABCC2* gene is located at chromosomal locus 10q24 and consists of 32 exons (31 coding exons) spanning 69 kb. It encodes an adenosine triphosphate (ATP)-binding cassette transporter protein, which is responsible for intracellular glucuronidation and the conjugation of GSH with clinically important drugs. The protein encoded by *ABCC2* is a member of the multidrug resistance like protein subfamily, which is involved in resistance to various drugs [17, 18]. ABCC2 protein is involved in biliary transport and is expressed in the canalicular (apical) part of hepatocytes. Its substrates include anticancer drugs, such as vinblastine; therefore, it appears to contribute to drug resistance in mammalian cells. In this study, we did not observe significant correlations between rs717620 variants and responses to different NAC chemotherapy regimens, including FOLFOX, SOX, XELOX, and XP, perhaps suggesting that it non-selectively targets these anticancer drugs. In *ABCC2* knockout mice, ABCC2 deficiency was associated with an increase in erythromycin metabolism [19]. In the same study, homozygosity for a reduced-function variant at rs717620 was also correlated with an increase in erythromycin metabolism in a cohort of 108 human subjects [19]. *ABCC2* is expressed in many tumor tissues, and tumor cells that overexpress *ABCC2* might acquire multidrug resistance [20]. For example, ABCC2 mRNA levels are positively correlated with cisplatin resistance in colorectal carcinoma [21].

Numerous SNPs have been identified in *ABCC2* [22–24]. Because of its importance in determining the disposition of anionic drugs and the pharmacological and/or adverse effects of substrate drugs, correlations between *ABCC2* genotypes and phenotypes require further study [22]. Among the many *ABCC2* SNPs, C-24T (promoter), G1249A (exon 10), and C3972T (exon 28) SNPs are relatively common [24, 25]. The G1249A polymorphism is a G > A base change that results in the amino acid substitution of Ile for Val at 417, and C3972T is a ‘silent’ mutation at 1,324 (Ile1324Ile). Several studies have suggested that these SNPs are associated with altered *ABCC2* expression or function [23, 26]. Besides promoting the export of glutathione-conjugated platinum, upregulated *ABCC2* expression also decreases the formation of platinum-DNA adducts and reduces G2-arrest in cisplatin-resistant cell lines [27]. However, one study found that ABCC2-24C > T did not affect DNA-protein binding or mRNA stability. Consistent with our results, Sun et al reported that mutant alleles in the promoter (C-24T) were related to the sensitivity of NSCLC to therapeutic agents, while SNPs in exons 10 (G1249A) and 28 (C3972T) were not associated with response to chemotherapy [15]. Other mechanisms may help explain these conflicting results. For example, most ABCC2 substrates are also transported by OATP2 (organic anion transporting polypeptide); therefore, *ABCC2* SNPs and inter-individual differences in OATP2 expression may need to be considered together.

Taken together, the present findings suggest that *ABCC2* C-24T polymorphic status may predict treatment responses and outcomes in advanced-stage GC patients, therefore facilitating the personalization of treatment strategies. This finding is consistent with previous reports on the potential role of *ABCC2* in drug resistance [13, 14]. Future studies are needed to confirm these results. Due to limited sample sizes, there may not have been significant statistical power to detect other factors that contribute to OS in GC patients. Furthermore, Eastern Asian populations may harbor ethnicity-specific variants in other candidate genes that contribute to drug resistance in GC patients in addition to the 23 SNP markers evaluated here. Because rs717620 is located in the 5′UTR of the *ABCC2* transcript, the −24C > T variant may increase *ABCC2* expression, especially in tumor tissue; this may partly explain the resulting inhibition of drug resistance and improved responses and outcomes associated with this SNP. We intend to conduct future validation studies to measure predictive and prognostic factors associated with this SNP. Larger sample sizes and prospective studies, independent collection of clinical outcome and genotyping data, tumor tissue expression analysis, and *in vivo* functional studies are needed to confirm our results and to clearly characterize the underlying mechanisms.

## MATERIALS AND METHODS

### Patients

The records of 103 patients with advanced GC who underwent surgery in the Department of Surgical Oncology at the Beijing Cancer Hospital (Beijing, China) between June 2008 and December 2013 were retrospectively reviewed. TNM stages were determined according to the 7th American Joint Committee on Cancer (AJCC) TNM system. All patients were monitored via inpatient follow-ups and outpatient records. OS was defined as the interval between the diagnosis of GC and GC-related death or the final visit. As of the most recent follow-up in April 2016, the median follow-up duration was 29.3 months (range: 5.4–82 months; Supplementary Table S1). A total of 33 (32.0%) patients died. Other relevant clinical features are summarized in Table 1. All patients provided informed consent. This study was reviewed and approved by the Institutional Review Board of the Beijing Cancer Hospital, and all experiments were conducted in accordance with the guidelines and regulations of the Institutional Review Board.

### Histopathological response evaluation

Resected specimens were processed in a highly standardized manner and histopathological analysis was conducted independently by two experienced pathologists. Postoperative pathologic TNM stages (ypTNM staging) were determined according to the Union for International Cancer Control (UICC), 6th Edition. The tumor regression grade (TRG) scoring system was applied to evaluate histopathologic regression in primary tumors [16]. TRG was scored as follows: 1a, no residual tumor/tumor bed; 1b, < 10% residual tumor/tumor bed; 2, 10–50% residual tumor/tumor bed; 3, > 50% residual tumor/tumor bed. Patients with less than 10% residual tumor were classified as responders.

### SNP selection

A set of 23 SNPs known to be related to platinum/5-Fu-based drug resistance was chosen from the Pharmacogenetics and Pharmacogenomics Knowledge Base (pharmGKB) database (Supplementary Table S2).

### DNA samples

Blood samples were obtained from all 103 patients. DNA was extracted from peripheral blood cells using the QIAamp^®^ DNA Blood Midi Kit (Qiagen, Hilden, Germany). All genotypes were examined using a SNaPShot^TM^ assay, polymerase chain reaction (PCR) amplification, and sequencing.

### SNP genotyping

Single nucleotide variant genotyping was performed using a SNaPShot™ SNP multiplex genotyping assay (ABI PRISM^®^ SNaPshot™ Multiplex Kit, Life Technologies, Grand Island, NY, USA) according to the manufacturer's instructions. Genetic regions flanking each genetic variant were co-amplified using 2 multiplex PCR primer pools. PCR amplification was performed in a total volume of 15 μL containing 50 mM KCl, 10 mM Tris-HCl (pH 8.3), 2 mM MgCl_2_, 300 μM of each dNTP, 0.5 U of KAPA Taq HotStart DNA Polymerase (Kapa Biosystems), 15 pmol of each primer, and 50 ng of gDNA. A standard touchdown PCR protocol was used. The primer sequences for the SNaPShot™ reaction are available upon request. Capillary electrophoresis was performed on an ABI^®^ 3130 Genetic Analyzer (Life Technologies, Inc.) and data were collected using GeneMapper^®^ version 4.0 software (Life Technologies, Inc.).

### Statistical analysis

Stringent quality control (QC) procedures were employed for each SNP, including the calculation of Hardy-Weinberg equilibrium (HWE) using Fisher's exact test [28]; allele frequencies were compared to reference samples such as the Han Chinese in Beijing, China (CHB) cohort in the 1000 Genomes Project (1 KG) or HapMap [29]. Associations between various genetic models (i.e., additive, dominance) and clinicopathologic features were quantified using the chi-square test for categorical variables and Student's *t*-tests for continuous data. Associations between genetic variants and clinicopathological features and OS were estimated using the Kaplan-Meier method, and comparisons among different groups of patients were performed using the log-rank test. All statistical analyses were conducted using SPSS version 16.0 for Windows (SPSS Inc., Chicago, IL, USA).

## SUPPLEMENTARY MATERIALS




