# Supplementary material for: ABCC2-24C > T polymorphism is associated with the response to platinum/5-Fu-based neoadjuvant chemotherapy and better clinical outcomes in advanced gastric cancer patients
Source: Oncotarget. 2016 Jul 30;7(34):55449–57. doi: 10.18632/oncotarget.10961 (PMC5342428; doi:10.18632/oncotarget.10961)
Supplement: Supplementary file 1 [file oncotarget-07-55449-s001.pdf]

ABCC2-24C > T polymorphism is associated with the response to platinum/5-Fu-based neoadjuvant chemotherapy and better clinical outcomes in advanced gastric cancer patients

Supplementary Materials

Supplementary Table S1: The clinical and genotype information of the patients.
See Supplementary_Table_S1.

Supplementary Table S2: Genetic markers evaluated in present study

Chromosome	Genes	db SNP	Alleles((on + chromosomal strand)	Amino Acid Translation	Position
3	UMPS	rs1801019	G > C	Gly213Ala	124456742
1	MTHFR	rs1801131	T > G	Glu347Ala	11794419
1	MTHFR	rs1801133	G > A	Ala140Val	11796321
1	DPYD	rs1801158	C > T	Ser534Asn	97515865
1	DPYD	rs1801159	T > C	Ile543Val	97515839
1	DPYD	rs1801160	C > T	Val732Ile	97770920
1	DPYD	rs1801265	A > G	Cys29Arg	97883329
1	DPYD	rs1801266	G > A	Arg235Trp	98157332
1	DPYD	rs1801267	C > T	Arg886His	97564154
1	DPYD	rs1801268	C > A	Val995Phe	97544627
1	DPYD	rs3918290	C > T	3'UTR	97915614
1	DPYD	rs17376848	A > G	Phe632Phe	97915624
1	DPYD	rs55886062	A > T, A > C	Ile560Asn,Ile560Ser	97981343
1	DPYD	rs67376798	T > A	Asp949Val	97547947
1	DPYD	rs72549306	C > A	Val335Leu	98058899
1	DPYD	rs78060119	C > A	Glu386null	98039499
1	DPYD	rs80081766	C > T	Arg21Gln	98348908
19	XRCC1	rs25487	T > C	Gln399Arg	44055726
7	ABCB1	rs1045642	A > T, A > G	Ile1145Ile	87138645
10	ABCC2	rs717620	C > T	5'UTR	101542578
11	GSTP1	rs1695	A > G	Ile105Val	67352689
19	ERCC1	rs3212986	C > A	Gln504Lys	45912736
1	DPYD	rs2297595	T > C	Met166Val	98165091
